# Evaluation of Efficacy of Long-term Growth Hormone Therapy in Patients with Hypochondroplasia

**DOI:** 10.4274/jcrpe.0043

**Published:** 2018-11-29

**Authors:** Tuğba Çetin, Zeynep Şıklar, Pınar Kocaay, Merih Berberoğlu

**Affiliations:** 1 Ankara University Faculty of Medicine, Department of Pediatric Endocrinology, Ankara, Turkey

**Keywords:** Hypochondroplasia, growth hormone therapy, skeletal dysplasia

## Abstract

Hypochondroplasia is a cause of disproportionate short stature and characterized by minor clinical manifestations. The aim of this study was to evaluate the efficacy of long-term growth hormone (GH) therapy in hypochondroplastic cases with inadequate response to GH stimulation tests. In this study, six patients who had a height standard deviation score of -3.43 before the treatment and a mean age of 7.42 years and who had received GH treatment at a dose of 0.2 mg/kg/week for a mean period of 4.45 years were evaluated. A good response was found in the first year of treatment, but this increase was not found to be sufficient for the patients to achieve an adequate final height.

**What is already known on this topic?**

Children with hypochondroplasia were given recombinant human growth hormone (GH) treatment in a limited number of studies. In these studies, treatment doses of GH and height gain were not uniform.

**What this study adds?**

This study presents the results of long term follow up of patients with hypochondroplasia by clinical, radiological and genetic examination and evaluates their response to growth hormone (GH) treatment. The study shows that high-dose GH therapy may be required for patients with skeletal dysplasia.

## Introduction

Hypochondroplasia is a common skeletal dysplasia that has an autosomal dominant inheritance and clinical manifestations of which can become evident over time. The patients may present with disproportionate short stature, macrocephaly, lumbar lordosis, rhizomelic and mesomelic shortness and brachydactyly (1,2). The patients may appear as normal or almost normal in early childhood and the clinical manifestations may not be evident until puberty. Final height has been reported to be 146±4.9 cm for males and 137.6±6.3 cm for females (3). Diagnostic radiological findings include decreased interpedicular distance between L1 and L5 and short lumbar pedicles (4). Hypochondroplasia is frequently due to the *FGFR3* mutation located at 4p16,3. However, mutations may not be observed in all hypochondroplasia patients. Children with hypochondroplasia were given recombinant human growth hormone (rhGH) treatment in a limited number of studies (5). In these studies, treatment doses of GH and height gain were not uniform. Very few data pertaining to the GH-insulin-like growth factor-1 (IGF-1) axis has been mentioned in the reported studies and very few studies have reported final height results following GH treatment.

In this study, we aimed to evaluate response to GH treatment in patients who had a definite diagnosis of hypochondroplasia by clinical, radiological and genetic examination, who also met the criteria for growth hormone deficiency and who received rhGH replacement therapy.

## Methods

In this study, we evaluated the growth of patients with hypochondroplasia who were followed in our clinic between the years 2000 and 2017 and who showed an inadequate response to growth hormone stimulation tests. A Harpenden stadiometer (Holtain Ltd, Crymych, Dyfed) was used in height measurements. Pubertal staging was done according to Tanner/Marshall criteria and bone ages were evaluated using the Greulich and Pyle (6) bone age atlas (7,8). The diagnosis of hypochondroplasia was made by clinical anthropometric evaluation (height, sitting height, upper/lower segment ratio etc.), presence of specific radiological findings (decreased interpedicular distance in the vertebrae, a short square iliac bone, extension in distal fibula) and demonstration of *FGFR3* gene mutation when possible. Presence of any affected parents in the family history supported the diagnosis. In all cases rhGH treatment was given at a dose of 0.2 mg/kg/week. Serum IGF-1 and IGF binding protein-3 concentrations, pubertal progression, change in upper/lower segment ratio, bone age progression rate and the achieved final height were assessed in the follow-up of the cases. All participants’ families gave informed consent and the study protocol was approwed by Ankara University Ethic Committe (approval number: 15-638-15).

## Results

Six cases (1 male/6 females) with an initial mean age of 7.08±3.2 years and one in puberty were included in the study. Before the treatment, mean annual height velocity of the patients was 3.96±0.83 cm, height standard deviation (SD) score (SDS) was -3.86±0.75, bone age was 4.84±2.97 years, upper segment/lower segment ratio was 1.34±0.2, and specific radiographic findings were present in all cases (Table 1). In the first year of growth hormone therapy, height velocity ranged between 6.9 and 10 cm (mean 8.4±1.29 cm) and it decreased to a mean of 5.5±1.76 cm in the third year and to 5.3±1.8 cm in the fourth year. In the first year of treatment, mean height SDS was -3.35±0.78, and Δ height SDS of +0.5 was found to decrease gradually in the following years. In the last follow-up of the cases when they had been on growth hormone therapy for a mean period of 4.45±1.3 years; their mean age was 12.48±3.19 years, mean height SDS -3.2±1.2 Δ height SDS 0.66±1.2 bone age 12.9±3.5 years and IGF-1 SD 1.34. Four of the patients were pubertal, with a mean puberty onset age of 10.66±1 years and achieved their final height. In these four patients, final height SDS was -3.57±1, Δ height SDS was +0.26±1.19. The upper/lower segment ratio did not change significantly and there was no increase in disproportionality. During treatment, IGF-1 levels did not exceed +2 SDS and remained within the confidence interval (Table 2).

## Discussion

The literature data on the use of growth hormone in skeletal dysplasias are scarce except for in achondroplasia, and usually short-term treatment results have been reported (9). Due to the genetic heterogeneity and phenotypic diversity of the diagnostic features, different conclusions regarding treatment efficacy were reported. Activating mutations of *FGFR3*, which have an effect on the negative regulation of cartilage growth are encountered in hypochondroplasia and achondroplasia (10). The N540K mutation is also seen in hypochondroplasia and is associated with more severe shortness and disproportion. However, mutations are not observed in all hypochondroplasia patients (11,12). In one of our cases, 1612 A>G (p.Ile538Val) heterozygous mutation in the *FGFR3* gene was detected.

In children with achondroplasia, with growth hormone treatment, a transient increase in height velocity without any effects on final height is observed, and consequently, the use of routine recombinant growth hormone in achondroplasia is not recommended (13). In children with hypochondroplasia, there is no placebo-controlled study on the effects of growth hormone treatment on adult height. Previous studies have shown that the use of growth hormone improves the increase in rate of height velocity in children with hypochondroplasia, especially at puberty (14,15). In a study by Pinto et al (16) in 2014, 40 patients were followed up without treatment until they achieved their final height, and these patients were compared with 19 patients with hypochondroplasia who were given 0.057 mg/kg/day growth hormone. In the growth hormone treated group the height increase rate of 5.1±0.3 cm/year at the beginning of treatment, reached 8.1±1.9 cm/year in the first year, 6.2±1.7 cm/year in the second and 4.8±2.2 cm/year in the third year. Similar to other reports, the best response was noted in the first year of treatment and decreased in the following years.

In a meta-analysis published in 2015, effects of growth hormone therapy was evaluated in hypochondroplastic cases. A total of seven publications and 113 patients with rhGH treatment were included in this meta-analysis. Among these patients, 59.7% were male and most of them were pre-pubertal at the beginning of the treatment. The average growth hormone dose was 0.25 mg/kg/week. In all studies, adult height which was predicted at the beginning of the treatment was below the normal level and an increase in adult height after rhGH treatment over predicted final height was reported. In the meta-analysis of these seven studies, in the median post-treatment predicted adult height SDS, there was an increase of 0.414 for the first year, 0.530 for the second year and 0.609 for the third year. This increase was especially notable in the first year of treatment and continued to decline thereafter. It was observed that the highest rate of height increase under treatment was the first year, suggesting that the effect of treatment on adult height was related to the height achieved for the first year. In our study, at the end of the first year of growth hormone treatment, delta height SDS was +0.51, suggesting that the response to growth hormone treatment was good. However, growth rates in subsequent years of follow-up decreased. IGF-1 levels remained within the confidence interval during treatment. In total, Δ height SDS remained at 0.21 and was interpreted as an insufficient response.

During the follow-up, we found that puberty started in the expected normal age range and that pubertal findings did not accelerate with GH treatment. Progression of bone age with GH therapy, which was reported in some skeletal dysplasia cases, is a finding that is important because of the effect on final height (17). In our cases, Δ bone age was found to be 6.6 years at the end of the treatment and the fact that there was not a significant increase was attributed to the accompanying GH deficiency.

An average of 28.5 cm height increase due to the treatment was observed in the four patients achieving their final height. After treatment, Δ height SDS was found to be +0.2. Upper/lower segment ratios during growth hormone therapy did not significantly worsen and no side effects were observed. The lack of change in disproportionality in patients with hypochondroplasia after GH therapy is a finding that suggests that the treatment does not have side effects. In patients with hypochondroplasia and GH deficiency, although rhGH therapy appeared to be effective, no improvement in final height was noted with the dose of 0.2 mg/kg/week.

In conclusion, this study showed that in GH deficient patients with hypochondroplasia, a standard dose of GH treatment was found to be sufficient to reach an increase in height just above 0.5 SD in the first year, which is acceptable, but not sufficient for the patients to achieve a sufficient increment in their final height. This finding supports the idea that high-dose GH therapy may be required for skeletal dysplasia, especially after the first year of rhGH therapy. Normal progression of puberty with a standard dose of GH therapy, lack of acceleration of bone age and skeletal disproportion and IGF-1 level remaining within the normal range are consistent with the idea that standard dose GH therapy is safe.

## Figures and Tables

**Table 1:**
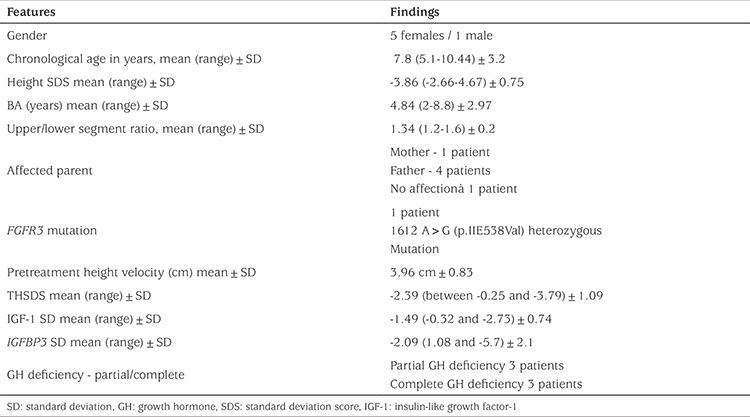
Features of the patients at presentation

**Table 2:**
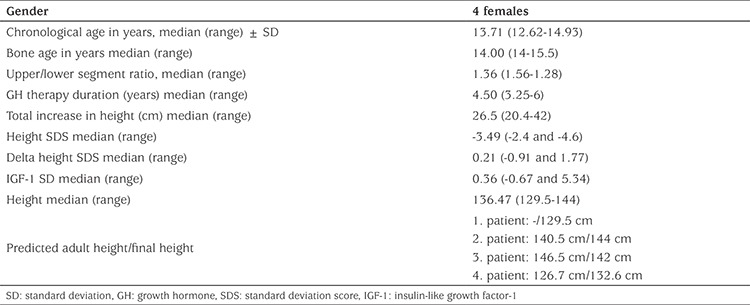
Characteristics of patients at cessation of treatment
